# Leveraging Personal Data to Enhance Machine Learning for Personalized Comfort Measurement

**DOI:** 10.3390/s26217077

**Published:** 2026-11-09

**Authors:** Richard Kaculi, Vittoria Cipollone, Nicole Morresi, Serena Serroni, Gian Marco Revel

**Affiliations:** 1 Department of Industrial Engineering and Mathematical Sciences, Università Politecnica delle Marche, 60121 Ancona, Italy; v.cipollone@pm.univpm.it (V.C.); s.serroni@pm.univpm.it (S.S.); gm.revel@staff.univpm.it (G.M.R.); 2 Department of Engineering and Science, Universitas Mercatorum, 00143 Rome, Italy; nicole.morresi@unimercatorum.it

**Keywords:** personalized thermal comfort, personalized comfort models, machine learning, wearable sensors, indoor environmental quality, thermal sensation vote, global sensation vote, occupant-centric monitoring

## Abstract

This study proposes a non-invasive experimental setup that integrates environmental and physiological sensors with personal parameters (age, gender, and Body Mass Index (BMI)) to measure the perceived thermal comfort of occupants via a Machine Learning (ML) framework. An experimental campaign involving 23 participants was conducted across cold (17–20 °C), neutral (22–24 °C), and warm conditions (26–28 °C). Data collected combined environmental measurements, continuous wearable physiological signals, subjective Thermal Sensation Votes (TSVs), and a multidimensional Global Sensation Vote (GSV). Evaluating six distinct datasets via a Leave-One-Subject-Out (LOSO) cross-validation strategy revealed that the value of personal data depends heavily on the underlying sensor architecture. While integrating personal data introduced slight noise in purely environmental linear models (decreasing accuracy by 4.77%), it yielded significant quantitative increments as sensor complexity shifted toward the individual, boosting accuracy by 2.03% in physiological setups and a remarkable 13.6% in bare-minimum sensor networks. Overall predictive performance reached an accuracy of 73% using a Support Vector Machine (SVM-LIN) for environmental configurations, while an XGBoost model achieved a robust 68% accuracy in a highly reduced, low-cost sensor setup by leveraging personal context. The innovative aspect of this research is the simultaneous use of sensors to monitor both physiological and environmental parameters, which, when combined with subjective feedback and personal data, significantly increases the prediction of TSV in practical cases. This integrated approach suggests prospective applications for vulnerable categories whose thermoregulatory responses deviate from standardized models, though it requires future empirical validation among these specific demographics. Overall, the integration of personal parameters into data-driven models contributes to generalizing individual physiological differences and supports the development of more effective, human-centric IoT monitoring systems.

## 1. Introduction

Indoor environmental quality (IEQ) plays a pivotal role for occupant health, cognitive efficiency, and overall building sustainability. While humans spend approximately 90% of their lives indoors, the measurement of the conditions that ensure their well-being remains dangerously approximate [1]. Poor indoor conditions go beyond simple discomfort, affecting both productivity and health, and are often associated with higher rates of Sick Building Syndrome and increased stress levels. Conversely, the accurate measurement and maintenance of thermal comfort is a catalyst for cognitive performance. For example, research in real homes shows that maintaining favorable temperatures and air quality substantially improves people’s scores on memory and attention tasks [1,2]. At the same time, buildings account for roughly 30% of global energy use and a similar share of CO_2_ emissions, so meeting tight carbon targets while keeping occupants comfortable is a major challenge to contribute to the EU sustainability goal envisioned for 2050 [3]. Recent evidence suggests that shifting from environmental conditioning to Personalized Thermal Comfort (PTC) systems can unlock energy savings between 20% and 37%, provided that the system can accurately measure when and where comfort is actually required [3,4].

Traditionally, researchers, constructors, and service providers rely on the Predicted Mean Vote (PMV) model as a standard for estimating thermal comfort. Developed from Fanger’s heat balance equations and calibrated in static climate chamber conditions, this model estimates the thermal sensation of a population under controlled environments. However, a key limitation of Fanger’s model is that the PMV represents the statistical mean thermal sensation of a group, not the actual thermal sensation experienced by an individual subject. The PMV index itself is expressed on a seven-point scale ranging from −3 (cold) to +3 (hot), with 0 representing thermal neutrality. The contemporary literature increasingly highlights the approximate representation of this approach for measuring individual well-being [5]. PMV fails to capture the specific physiological responses of real occupants by averaging human diversity. Moreover, Fanger’s model is not always reliable in real-world scenarios or when applied to specific population groups, such as elderly people or children, whose thermoregulatory responses differ from those assumed in the original model. Consequently, when applied in dynamic real-world settings, the PMV model predicts occupants’ actual thermal sensation with less than 34% accuracy [6,7,8].

Building on the limitations of Fanger’s model, Personalized Comfort Models (PCMs) have emerged as a more adaptive approach to thermal comfort assessment. Unlike population-based models, PCMs aim to estimate comfort at the individual level by integrating subject-specific information [9]. In this framework, personal data, such as gender, age, and Body Mass Index (BMI), are treated as interfering factors in the thermoregulatory process. The importance of these personal parameters becomes even more evident when examining their specific physiological impacts. Gender, for instance, influences baseline thermal neutrality, with studies indicating higher neutral temperatures for females compared to males [10]. Age affects metabolic heat production and vascular responsiveness, altering thermal sensitivity [11]. Concurrently, BMI serves as a standardized estimate of body composition and a proxy for passive insulation, influencing both skin temperature and perceived warmth [12].

These factors do not directly define comfort conditions but modulate the relationship between the environment and human perception [13]. Precisely for this reason, their inclusion provides meaningful context that allows PCMs to interpret thermal responses more accurately.

When explicitly coupled with dynamic physiological data, such as skin temperature, heart rate, heart rate variability, and activity level, these inputs provide a comprehensive description of the body’s interaction with its surroundings [9,13]. Ultimately, combining personal data with real-time physiological indicators provides the meaningful context necessary for PCMs to move beyond generalized assumptions and accurately interpret human thermal responses under real-world conditions.

A key advantage of PCMs lies in their ability to incorporate human feedback for model calibration and continuous adaptation [14,15]. Subjective feedback, such as the Thermal Sensation Vote (TSV), provides direct insight into occupants’ perceived comfort. The TSV follows the same seven-point scale as the PMV (from −3 cold to +3 hot), but, being categorical, it reflects the actual sensation experienced by the individual rather than a predicted mean value [16]. In parallel, objective indices such as the Indoor Global Comfort Index (IGCI) enable a comprehensive evaluation of indoor environmental quality by integrating multiple comfort domains, including thermal, acoustic, visual, and air quality comfort, into a single metric [17,18,19]. By combining physiological measurements, subjective responses, and holistic indices like the IGCI, PCMs can better capture both perceived and measured aspects of comfort [9,20]. This integrated approach allows models to iteratively adapt to user-specific preferences, further reinforcing the role of personal data in improving prediction accuracy and enabling a truly human-centric assessment of indoor environmental quality [21].

To move from generalized estimations toward tailored individual measurement, recent research has increasingly adopted Machine Learning (ML) approaches [22,23]. Within this framework, the measurement system evolves from a static physical sensor into an adaptive computational model capable of fusing environmental, physiological, and personal data. In this context, ML algorithms act as powerful tools to extract meaningful patterns from heterogeneous inputs, demonstrating that the inclusion of personal data is not merely supplementary but central to improving model performance.

Among ML techniques, tree-based ensemble methods currently represent the state-of-the-art for processing categorical comfort data. For instance, Random Forest (RF) models combined with SHAP-based feature selection have demonstrated significant improvements in prediction performance, with reported accuracy in predicting thermal sensation increasing from 90% to 95% when personal data are explicitly incorporated [23]. Similarly, XGBoost models integrating metabolic rate and clothing insulation data have achieved an R^2^ of 0.94 and accuracy levels up to 96.7% in predicting thermal sensation, outperforming traditional approaches such as Support Vector Machines (SVMs), which typically plateau around 81% [23]. These results consistently highlight that models leveraging personal data outperform those based solely on environmental inputs.

While these approaches perform well on static datasets, Deep Learning techniques, particularly Long Short-Term Memory (LSTM) networks, enable the modeling of temporal dynamics in thermal comfort. Applications in sensitive populations, such as the elderly, have achieved accuracy levels of approximately 90% in predicting thermal sensation by capturing the sequential evolution of physiological signals, confirming that comfort is a time-dependent process influenced by prior exposure and adaptation [5].

In addition to standard predictive ML techniques, the recent literature has explored optimization-based approaches to achieve individualized thermal comfort inference. For instance, Genetic Algorithms (GA) have been successfully applied to directly address individualized thermal-comfort modeling, with studies demonstrating that personalizing parameters like metabolic rate via GA can achieve thermal comfort estimation accuracies of 98%, compared to just 73% when relying on standard ASHRAE tables [24]. These GA-based methodologies typically operate by taking generalized analytical equations (such as Fanger’s PMV) and dynamically tuning their coefficients based on continuous individual occupant feedback. This allows the model to learn a highly specific comfort profile tailored to a single user over time. While highly effective at individualization, these approaches fundamentally differ from the methodology proposed in this study. GA models generally rely on an underlying theoretical equation structure and require a continuous optimization feedback loop tailored to a specific occupant. In contrast, our proposed methodology is purely data-driven and seeks to generalize personalization across a population. Instead of mathematically evolving a unique model for every individual user, our approach bypasses theoretical equations entirely to fuse multi-domain raw data, specifically, personal data (age, gender, BMI) combined with real-time physiological signals and environmental data. By using these personal data to add valuable information within a classification framework, the model naturally contextualizes baseline physiological differences across diverse users without the need for occupant-specific mathematical optimization.

Despite the critical importance of IEQ for human health and building performance, its accurate assessment remains a persistent challenge. While modern sensing technology has advanced, the frameworks used to interpret this data remain anchored in historical models that prioritize group averages over individual reality [25,26]. The reliance on Fanger’s PMV represents a significant bottleneck in this regard; as a metric, it collapses human diversity into a statistical mean, effectively ignoring the physiological nuances that define actual comfort. This results in a dangerous approximation of indoor conditions where predictive accuracy frequently falls below 34% in real-world scenarios, when PMV is compared to the actual occupants’ thermal sensation. The primary limitation in current research lies in how personal data are integrated into PCMs. Although modern ML models for thermal comfort estimation frequently utilize personal data (e.g., age, gender, and BMI) as inputs, the state of the art fails to systematically assess their added value. The main contribution of this paper is explicitly measuring exactly how much personal data improves thermal comfort predictions. Furthermore, this study highlights a non-invasive experimental setup using sensors to monitor both physiological responses and environmental conditions, alongside subjective feedback and personal data, to improve the prediction of TSV in real-world scenarios. While this study is specifically validated for young adults in an office environment, this multi-sensor approach lays the methodological groundwork for potential future applications in vulnerable categories, such as the elderly or individuals with compromised thermoregulation. Because traditional, generalized population models frequently fail to capture individual comfort perceptions in these demographics, future dedicated experimental campaigns could adapt this methodology to address their specific physiological needs.

This study achieves this through three primary objectives. First, it systematically compares ML-based TSV approaches across six distinct datasets, alternating combinations of environmental, physiological, and personal data to definitively verify whether personal inputs improve prediction accuracy. Second, rather than treating age, gender, and BMI as mere supplementary variables, a targeted feature-importance analysis isolates and quantifies their precise impact on model accuracy. Finally, moving beyond single-domain assessment, this work incorporates a descriptive statistical analysis of the Global Sensation Vote (GSV) grounded in the IGCI framework. Rather than serving as an ML predictive target, this holistic metric, which integrates thermal, air quality, acoustic, and visual comfort into a unified scale, is evaluated to quantitatively demonstrate the direct dependency between targeted thermal neutrality and overall occupant satisfaction.

## 2. Materials and Methods

As shown in Figure 1, this study presents a measurement framework for estimating perceived thermal comfort (i.e., TSV) via ML. The methodology is based on the acquisition of environmental sensor data, physiological signals, personal data, and human perceptions. Environmental variables were obtained from calibrated indoor sensors, while physiological data were continuously monitored through wearable sensing devices. The human perceptions were collected by a questionnaire provided through a dedicated web page, enriched by personal data such as age, gender and BMI. Following initial integrity checks to verify the consistency and coherence of the collected measurements, as well as synchronization and preprocessing, the multi-domain measurements were consolidated into a unified dataset. Statistical and feature analyses were conducted to quantify correlations and identify the most informative parameters to use as input features for the ML models. To evaluate the contribution of different sensing configurations, the unified dataset was partitioned into six sub-datasets, each representing a distinct combination of measurement parameters. These sub-datasets were used to train and test the ML models. Finally, the feature importance analysis quantifies the value of information of each parameter in each sub-dataset configuration.

### 2.1. Participants

The study involved 23 participants (11 women, 12 men) aged 20–35 years (mean 28.3 ± 2.4 years), selected from university staff and researchers to ensure a homogeneous sample in terms of age, occupation, and lifestyle. Mean weight and height were 69.1 ± 15.6 kg and 1.73 ± 0.09 m, with a mean BMI of 22.9 ± 3.9 kg/m^2^. Participants primarily belonged to academia, including PhD students, postdocs, researchers, and professors, representing sedentary office workers. To ensure the objectivity of the tests, participants were not required to follow any specific clothing protocol. All participants provided written informed consent for the use of their personal data and were fully informed about the purpose of the study. All experimental procedures were conducted in accordance with the WMA Declaration of Helsinki and with the ethical standards established by the Ethical Committee of Università Politecnica delle Marche (UNIVPM), Ancona, Italy.

### 2.2. Measurement Setup

The experimental trials were conducted from February 5th to 18th,2025, across three distinct indoor thermal conditions: cold (17–20 °C), neutral (22–24 °C), and warm (26–28 °C). The measurement campaign took place in a dedicated office space located within the Department of Industrial Engineering and Mathematical Sciences (DIISM) at Polo Monte Dago of UNIVPM, Ancona. The test room (2.90 m × 4.80 m) was configured to enable comprehensive and integrated data collection as reported in Figure 2. The measurement setup included a sensor network for assessing indoor environmental conditions, and a set of wearable and minimally invasive devices for physiological signal acquisition. The users’ subjective perceptions (i.e., TSV) were collected through a dedicated online questionnaire, as will be explained in the following paragraphs.

#### 2.2.1. Environmental Parameters

During the experimental phase, continuous monitoring of environmental parameters was carried out to objectively characterize the microclimatic conditions experienced by participants. The selected parameters align with the established literature and major standards on indoor comfort and environmental quality [27]. Data were collected using the Netatmo Smart Indoor Air Quality Monitor from Netatmo company, France (Figure 3), positioned within the testing environment to perform continuous monitoring. This device provided multiple environmental parameters, including the following:

- Air temperature (T, °C);
- Relative humidity (RH, %);
- CO_2_ concentration (ppm);
- Ambient noise levels (dB).

Measurement resolution and accuracy for these variables are reported in Table 1. Spot measurements using thermocouples were conducted to monitor the external temperature (Tout). Measuring Tout is crucial because outdoor climates significantly dictate the indoor thermal environment and play a fundamental role in shaping occupants’ thermal sensation and adaptation expectations [28]. T was measured near the workstation to accurately reflect actual occupant conditions, while RH was monitored in real-time to capture its impact on thermal perception.

#### 2.2.2. Physiological Parameters

Physiological parameters were measured using wearable devices selected for their minimal interference with daily activities. These data complemented environmental and subjective measures, providing an integrated view of individual responses to thermal stimuli:

- Forehead temperature (Tf): Continuously measured with an iButton sensor from Analog Devices company, Dallas, TX, USA (Figure 3) secured by a headband, offering reliable, non-invasive monitoring of a thermally sensitive skin area.
- Peripheral skin temperature (Ts) and heart rate: Recorded using the Empatica EmbracePlus from Empatica Inc. company, Cambridge, MA, USA (Figure 3), a wrist-worn device equipped with a 64 Hz photoplethysmographic (PPG) sensor.

#### 2.2.3. Personal Parameters

Personal parameters were collected through a form administered before and during the experimental sessions. Each participant completed a preliminary form recording demographic and individual data, including age, gender, weight, height, occupation, and physical activity habits. All data collection and processing procedures strictly complied with the General Data Protection Regulation (GDPR) to ensure participant privacy and data confidentiality.

During the experiments, standardized digital questionnaires were used to gather subjective indicators of thermal perception and environmental well-being. The primary index was the TSV, evaluated using the standard ASHRAE 7-point scale ranging from −3 (cold) to +3 (hot), with 0 representing thermal neutrality. Participants recorded their subjective TSV via the digital interface at predefined intervals. Complementary measures included the GSV. The GSV captures the user’s holistic subjective evaluation of the environment, requiring them to integrate their perception of thermal, visual, acoustic, and air quality factors, independent of instrumental measurements for each domain. The GSV was rated on a scale from 1 to 10, where 1 indicates poor overall comfort conditions, and 10 indicates excellent overall comfort. All questionnaires were designed for quick digital administration to minimize interference with participants’ activities.

### 2.3. Measurement Protocol

Upon arrival at the test room, each participant signed an informed consent form, which included a preliminary questionnaire on thermal sensation. Participants were then instrumented with physiological sensors: the Empatica E4 wristband worn on the non-dominant hand and an iButton sensor positioned on the forehead for continuous Ts and Tf measurement, and received instructions on the use of the web-based application for subjective data collection.

Once all physiological sensors were installed, the experimental session commenced. The entire test lasted a total of 38 min and was divided into two distinct phases, as shown in Figure 4. First, a 20-min acclimatization period began, during which participants remained seated at the workstation. To ensure continuous monitoring of thermal perception, subjective questionnaires were administered every 4 min via the web application, starting from the beginning of the acclimatization phase and continuing throughout the entire experimental session.

Following the initial acclimatization, the second phase began, wherein participants engaged in a standardized computer-based office activity lasting 18 min, while maintaining the same 4-min interval for questionnaire completion. This sampling rate allowed for granular tracking of both physiological and subjective transitions. Upon completion of the session, the operator verified correct data acquisition and reset all physiological sensors for the next participant.

### 2.4. Data Analysis and Feature Analysis

This section outlines the complete data preparation and analysis pipeline prior to ML modeling. It details how raw sensor and survey data were temporally synchronized, statistically evaluated to remove redundancies, and ultimately filtered to select the most relevant features for predicting thermal comfort.

#### 2.4.1. Dataset Creation

The preparation and structuring of the experimentally measured data represented an important phase of the work, as the quality and reliability of the subsequent statistical analyses and modeling activities depended directly on it. The process involved the integration of heterogeneous data sources, merging continuous raw data from environmental and wearable sensors with subjective responses and participant personal data. An important step was the temporal synchronization of these different measurement streams; the timestamps of the subjective questionnaires (TSV) served as the primary anchor points. Continuous physiological and environmental readings were aligned to these specific timestamps using the interpolation method, ensuring that user perceptions were directly mapped to the concurrent physical conditions. The unified dataset aggregates measurements across three experimental thermal conditions: cold (17–20 °C), neutral (22–24 °C), and warm (26–28 °C). Following data fusion, a quality control phase was conducted to verify data continuity and coherence through the visual inspection of temporal trends. Table 2 reports the measured variables divided by each category.

#### 2.4.2. Statistical Analysis

Statistical analysis was conducted before developing and implementing the ML models, with the purpose of performing data exploration and understanding the relationships between collected variables, identifying correlations and selecting the most relevant features for the prediction of TSV. The following indicators have been selected: (i) First, the Chi-Square Test was implemented for categorical variables to evaluate the existence of significant associations between observed categories. This test was used to highlight statistical dependence between TSV and GSV, as well as to validate demographic features. In accordance with recent feature selection protocols for thermal comfort modeling [29], the test statistic is calculated as in Equation (1):

(1) $$x^{2}=\sum_{i=1}^{n}\sum_{j=1}^{m}\frac{{\left(O_{\mathrm{ij}}-E_{\mathrm{ij}}\right)}^{2}}{E_{\mathrm{ij}}}$$

where *O_ij_* represents the observed frequency and *E_ij_* the expected frequency under the assumption of independence. This method ensures that only categorical variables with a statistically significant impact on thermal perception (e.g., *p* < 0.05) are retained for the modeling phase, a practice supported by recent behavioral studies in educational and industrial settings [30,31,32]. (ii) Secondly, Pearson’s correlation coefficient was calculated for quantitative variables to measure the linear relationship between pairs of variables. This step quantified the linear relationships between measured environmental, physiological, and subjective parameters. To mitigate severe multicollinearity and filter redundant features, variables exhibiting a high Pearson correlation (e.g., |*ρ*| ≥ 0.80) were evaluated to retain only a single representative parameter [32]. This prevents the destabilization of ML feature importance algorithms without artificially biasing the models.

The Pearson correlation coefficient (*r*) was computed using the standard formulation applied in recent sensor-based studies [29,33] as in Equation (2):

(2) $$r=\;\frac{\sum_{i=1}^{n}\left(x_{i}-\bar{x}\right)\left(y_{i}-\bar{y}\right)}{\sqrt{\sum_{i=1}^{n}{\left(x_{i}-\bar{x}\right)}^{2}}\sqrt{\sum_{i=1}^{n}{\left(y_{i}-\bar{y}\right)}^{2}}}\;$$

where *x_i_*, *y_i_* are the individual data points for each variable and $\bar{x}$, $\bar{y}$ represent the average (mean) of all the values collected for each variable. This analysis guided the selection of the most relevant variables for the predictive modeling phase [34].

#### 2.4.3. Feature Selection

The Pearson correlation coefficients between variables were utilized to identify the parameters most correlated with TSV, which is the label of the ML approaches explained in the next paragraphs. In accordance with the state of the art, *r* were stratified into three levels to guide feature selection: strong correlation (*r* ≥ 0.70), indicating a dominant predictive relationship where the variable explains a major proportion of the variance; moderate correlation (0.40 ≤ *r* < 0.70), suggesting a significant contributory factor that refines model sensitivity; and low correlation (*r* < 0.40), representing a negligible linear association.

### 2.5. ML Model Training and Testing

Following the preliminary statistical analysis, ML techniques were applied to develop predictive models capable of estimating the TSV based on collected environmental, personal and physiological data. To ensure a comprehensive evaluation, a diverse set of algorithms was implemented:

- **Linear Models:** Linear Regression (LR) and Support Vector Machines (SVM-LIN and SVM-RBF) [35].
- **Distance and Probabilistic Models:** K-Nearest Neighbors (KNN) and Naive Bayes (NB) [36].
- **Neural Networks (NN):** Multi-Layer Perceptron (MLP) [37].
- **Tree-Based Ensemble Methods:** Random Forest (RF), XGBoost (XGB), LightGBM (LGBM) and Gradient Boosting (GBC) [35,38].

To address the inherent class sparsity of a small dataset (647 total observations) and improve model stability, the original 7-point ASHRAE scale was mapped into a 3-class classification problem: Cold (TSV < 0), Neutral (TSV = 0), and Hot (TSV > 0) [39]. The original 7-class distribution contained very few instances at the extreme thermal sensations, which destabilizes model training. The 3-class aggregation resolves this severe imbalance, resulting in 182 observations for the Cold class, 286 for the Neutral class, and 179 for the Hot class.

To evaluate the models’ generalization capabilities to previously unseen occupants, a Leave-One-Subject-Out (LOSO) cross-validation strategy was implemented [32,40]. Instead of a random sample-level split, the dataset was partitioned at the participant level. During each iteration, all observations from a single participant were isolated as the test set, while the data from the remaining 22 participants constituted the training set. This process was repeated iteratively until every participant had been used exactly once as the test set.

All variables were standardized using a standard scaler, transforming the data to have a mean of zero and a standard deviation of one. The scaler was fitted exclusively on the training fold and subsequently used to transform the isolated test fold.

Given the reduced size of the dataset, hyperparameter selection was configured using default hyperparameters (Table 3). Performing a grid-based hyperparameter optimization on a small sample size might increase the risk of overfitting, forcing the model to memorize noise and individual participant particularities rather than learning generalized physiological and environmental relationships. Therefore, utilizing established baseline configurations provides a more authentic representation of each algorithm’s baseline discriminative power for this specific application.

Following the training and testing phases, the predictive performance of the ML classifiers was quantitatively evaluated using the related standard classification metrics: *Accuracy*, *Precision*, *Recall* and *F1-Score*. These metrics provide information, offering both a global measure of correct predictions and a robust assessment of the model’s discriminative power. *Accuracy* evaluates the overall ratio of correctly predicted observations to the total observations, providing a direct indication of the model’s general performance. It is defined as in Equation (3):

(3) $$\mathrm{Accuracy}=\frac{\mathrm{TP}+\mathrm{TN}}{\mathrm{TP}+\mathrm{TN}+\mathrm{FP}+\mathrm{FN}}\;$$

where TP (True Positives) and TN (True Negatives) represent the number of outcomes correctly predicted by the model, while FP (False Positives) and FN (False Negatives) denote the misclassified instances. The denominator represents the total number of samples in the dataset.

To provide a more in-depth evaluation, *Precision* and *Recall* were considered as well. *Precision* (or positive predictive value) quantifies the exactness of the model by measuring the proportion of true positive predictions among all positive predictions made, as in Equation (4):

(4) $$\mathrm{Precision}\;=\frac{\mathrm{TP}}{\mathrm{TP}+\mathrm{FP}}\;$$

*Recall* (or sensitivity) assesses the completeness of the model by measuring the proportion of actual positives that were correctly identified, as in Equation (5):

(5) $$\mathrm{Recall}\;=\frac{\mathrm{TP}}{\mathrm{TP}+\mathrm{FN}}\;$$

While *Accuracy* gives an immediate sense of precision, it can be misleading in cases of class imbalance. To address this, the *F1-Score* was calculated. The *F1-Score* is the harmonic mean of *Precision* and *Recall*. It is mathematically expressed as in Equation (6):

(6) $$F1-\mathrm{Score}=2\;\times \frac{\mathrm{Precision}\;\times \;\mathrm{Recall}}{\mathrm{Precision}+\mathrm{Recall}}=\;\frac{2\;\times \;\mathrm{TP}}{2\;\times \;\mathrm{TP}+\mathrm{FP}+\mathrm{FN}}$$

The *F1-Score* ranges between 0 and 1. Specifically, the values close to 1 indicate high classification performance with a balance between precision and sensitivity, whereas values near 0 suggest the model fails to effectively distinguish between classes.

Finally, class-wise performance and specific misclassification trends were visually analyzed through the generation of confusion matrices for each evaluated model.

Beyond global classification metrics, the robust evaluation of the added value of personal data required in-depth statistical testing [41]. Because the LOSO cross-validation strategy evaluates the model on independent subjects, the accuracy for each individual subject was recorded to calculate the mean accuracy and standard deviation (SD). This subject-level resolution provides insight into the model’s stability across different individuals. To estimate the true performance on the broader population, 95% confidence intervals (CIs) were calculated based on the standard error of the mean across the LOSO folds [42].

Finally, to isolate and statistically validate the impact of incorporating personal data, a paired statistical approach was utilized [43]. For each paired dataset configuration (e.g., environmental data only vs. environmental and personal data), the performance of the exact same ML algorithm, evaluated on the exact same LOSO partitions, was compared using a paired *t*-test. A *p*-value threshold of <0.05 was established to determine whether the performance variations observed upon adding personal data were statistically significant or merely due to random variance.

### 2.6. Feature Importance Assessment

Following model training, feature importance analysis was utilized to interpret the internal logic of the ML algorithms. In this domain, feature importance is a technique that assigns a quantitative score to each input variable, reflecting its relative contribution to the model’s predictive accuracy [44]. To evaluate the diverse set of ML algorithms on an equal footing, Permutation Feature Importance was employed. This technique calculates importance by measuring the decrease in a model’s performance when a single feature’s values are randomly shuffled, thereby breaking the structural relationship between that specific feature and the target TSV [45].

For each model and sub-dataset configuration, the permutation process was repeated 10 times on the standardized data to ensure the statistical stability of the estimations and reduce variance. To enable a direct comparison of feature weights across different algorithms and varying dataset dimensions, the raw importance scores were subjected to a strict normalization protocol. First, any negative importance values, which can occur when random shuffling coincidentally improves performance on small datasets due to chance, were clipped to zero to prevent analytical distortion. Subsequently, the remaining positive importance scores were normalized by dividing each feature’s score by the sum of all importances within that specific model and dataset configuration. This scaling ensured that the feature importances for any given configuration summed to exactly 1 (100%). By isolating these normalized weights, this method provides a standardized, proportional assessment of the “value of information” embedded in the environmental, physiological, and personal parameters across the six different sub-datasets.

## 3. Results

This section presents the findings derived from the experimental campaign and evaluates the efficacy of data-driven approaches for predicting personalized thermal comfort. The objective of this section is twofold: to uncover the relationships between different kinds of measurements and subjective human perception, and to validate the predictive capacity of various ML architectures under different data availability.

### 3.1. Preliminary Data Analysis Results

As outlined in the methodology, a preliminary statistical analysis was conducted to explore the collected data and identify correlations between monitored environmental parameters and subjective thermal comfort perception.

#### 3.1.1. Gender Differences

Figure 5 presents the distribution of TSV stratified by gender. The boxplot analysis reveals a distinct divergence in thermal perception between the groups. The female group (f) exhibits a median TSV situated within the negative range, with the range largely confined between −1 (slightly cool) and 0 (neutral). In contrast, the male group (m) displays a median TSV in the positive range, with a distribution skewed towards warmer sensations. These results indicate that under the experimental conditions, female participants reported a higher sensitivity to cooler temperatures and a general tendency toward thermal discomfort in cold environments. This tendency aligns with the established literature regarding gender-related physiological differences, such as lower metabolic rates and different Ts distributions in females compared to males.

#### 3.1.2. Personal Parameters

The impact of individual personal parameters, specifically BMI and age, on thermal sensation was analyzed separately for the Cold and Hot trials, as illustrated in Figure 6. In the Cold Trial (Figure 6, left), a consistent pattern emerged across both age demographics (20–30 and 30–40 years), where participants with a BMI < 22 Kg/m^2^ consistently reported lower mean TSV values compared to those with a BMI ≥ 22 Kg/m^2^. This trend suggests that individuals with lower body mass experienced the cold more intensely, likely attributable to reduced subcutaneous fat acting as thermal insulation. Conversely, the Hot Trial (Figure 6, right) revealed a more complex relationship; while the younger age group (20–30 years) generally reported higher TSVs closer to “slightly warm,” the older cohort (30–40 years) leaned toward neutrality. Notably, within the 30–40 years age group during the Hot Trial, participants with lower BMI reported the lowest thermal sensation scores, indicating a potential divergence in heat tolerance or thermal sensitivity linked to body composition. While the limited sample size necessitates caution in generalizing these findings, the data suggest that physiological characteristics, specifically the combination of gender and body composition, play a non-negligible role in subjective thermal assessment.

#### 3.1.3. Relationship Between TSV and GSV

The investigation into subjective thermal experience was expanded by analyzing the relationship between the TSV and GSV, utilizing their respective categorical scales. The Chi-Square test established a statistically significant dependency between the two categorical indices, evidenced by an extremely low *p*-value (<0.001). This confirms a strong relationship between an individual’s specific thermal sensation and their overall perception of comfort. As shown in Figure 7, a distinct pattern emerged: the optimal condition for TSV is the thermal neutral point (0), while the best for GSV in comfort is approaching 10. Visual inspection of the fitted data confirms this strong correlation, even if non-linear. Specifically, at TSV = 0, both the mean and median GSV peak at 8 (within an interquartile range of 7 to 9), representing the highest observed central tendency for comfort across the scale. This convergence clearly demonstrates the strong connection between feeling thermally neutral and reporting a high level of global comfort.

#### 3.1.4. Parameter Correlation and Dataset Configuration

The modeling process evaluated the impact of various parameter combinations on the predictive algorithms, following a gradual approach informed by the statistical analysis. Specifically, the Pearson correlation matrix (Figure 8) helped identify the parameters most correlated with TSV. The analysis confirmed a strong correlation between TSV and T (r = 0.73), highlighting the dominant linear association of ambient temperature with thermal perception. Peripheral Ts measured by the Empatica sensor also correlated with TSV (r = 0.55), indicating a statistical association with physiological responses. Similarly, Tf (r = 0.55) and CO_2_ concentration (r = 0.53) showed positive associations. It must be emphasized that these coefficients represent observational correlations rather than direct causal relationships; for instance, the positive association with CO_2_ suggests that deteriorating air quality frequently co-occurs with higher thermal sensation votes in this specific test environment, rather than establishing CO_2_ as a direct physiological driver of thermal discomfort. Furthermore, the analysis revealed a high collinearity between T and Tw, with a correlation of r = 0.93. In accordance with the feature selection protocol designed to mitigate multicollinearity, only a single representative parameter was retained to prevent artificial model bias. T was selected over Tw because air temperature remains the most monitored and fundamental baseline parameter in standard thermal comfort assessments [46].

While Tw is generally a better proxy for mean radiant temperature, its near-perfect synchronization with ambient air temperature in our experimental context ensures that its thermal variance is already structurally captured by T. Therefore, its omission preserves the mathematical robustness and interpretability of the evaluated ML models. Conversely, ambient noise levels demonstrated a negligible linear association with thermal perception (*r* = 0.13). Following the established feature selection methodology, Noise was evaluated in the initial comprehensive configurations but subsequently removed from the optimized, reduced datasets due to its lack of predictive power. Overall, these findings informed the selection of variables for the predictive models, leading to six dataset configurations: (1) environmental and personal data, (2) environmental data, (3) environmental, personal, and physiological data, (4) environmental and physiological data, (5) reduced environmental and reduced physiological data, and personal data, and (6) reduced environmental and physiological data (Table 4).

### 3.2. ML Results

This study evaluated the performance of several ML algorithms across six distinct input datasets to predict TSV using a LOSO cross-validation strategy. The results for *Accuracy*, *F1-Score*, *Recall*, and *Precision* for the best ML model for each configuration, alongside their statistical evaluations (mean, accuracy, standard deviation, 95% confidence intervals, and paired *t*-test *p*-values), are presented in Table 5.

**Dataset 1: Environmental and Personal Data**

The first configuration utilized a comprehensive set of environmental parameters (CO_2_, RH, T) and personal data (age, gender, BMI). In this setting, the SVM-LIN model demonstrated the strongest performance, achieving a mean *Accuracy* of 0.79 ± 0.2 (95% CI [0.6; 0.7]), an *F1-Score* of 0.69, a *Recall* of 0.70, and a *Precision* of 0.70. The regression analysis (Figure 9a) visually confirms this alignment, while the confusion matrix (Figure 10a) reveals the model’s classification spread across the mapped classes. Feature importance analysis (Figure 11a) identified T as the principal determinant with an overwhelming importance score of 0.93, followed by a minor contribution from CO_2_ concentration (0.04) and BMI (0.02). Other features, including RH (0.00), age (0.00), and gender (0.00), had negligible to zero impact on the model’s predictions.

**Dataset 2: Environmental Parameters Only**

By contrast, Dataset 2 relied exclusively on environmental parameters without personal data. The SVM-LIN model achieved a mean *Accuracy* of 0.73 ± 0.2 (95% CI [0.6; 0.8]), an *F1-Score* of 0.73, a *Recall* of 0.73, and a *Precision* of 0.73. Comparing this to Dataset 1 demonstrates a performance drop of −4.7% in accuracy when personal data was introduced. However, a paired *t*-test reveals this decrease is not statistically significant (*p* = 0.27). This confirms that for this specific linear algorithm, the addition of personal data had no actual effect on predictive performance, merely introducing slight, non-significant noise. The model relies entirely on ambient conditions (Figure 11d), with T completely dominating the feature importance at 0.96, followed by a marginal contribution from CO_2_ (0.04) and zero importance for RH (0.00). The confusion matrix (Figure 10d) reflects a strong bias toward correctly predicting ambient baseline thresholds without the added complexity of personal metadata.

**Dataset 3: Environmental, Physiological and Personal Parameters**

Dataset 3 aimed at enhancing the prediction of TSV by integrating physiological parameters (Tf, Ts, heart rate), combining these with both environmental and personal data. Here, the KNN algorithm proved most effective, achieving a mean *Accuracy* of 0.64 ± 0.2 (95% CI [0.5; 0.7]), an *F1-Score* of 0.64, a *Recall* of 0.64, and a *Precision* of 0.64. The confusion matrix (Figure 10b) shows the model capturing the ‘Comfort’ class but exhibiting slightly higher misclassifications between adjacent thermal states compared to the simpler Dataset 2. Feature importance (Figure 11b) highlights a distinct shift in predictive influence, exhibiting a highly balanced distribution among variables: T remains the highest (0.15), closely followed by physiological parameters Ts (0.13) and Tf (0.12). Other factors also show significant, evenly distributed contributions, including CO_2_ (0.13), RH (0.11), BMI (0.10), heart rate (0.10), gender (0.08), and age (0.08).

**Dataset 4: Environmental and Physiological Parameters Only**

Dataset 4 included environmental and physiological parameters but omitted personal data. The highest performance in predicting TSV was reached by the KNN model with a mean *Accuracy* of 0.63 ± 0.2 (95% CI [0.5; 0.7]), an *F1-Score* of 0.63, a *Recall* of 0.63, and a *Precision* of 0.63. Comparing this to Dataset 3, the reintroduction of personal data yielded a marginal increase in predictive metrics, but statistical analysis confirms this slight variation is not significant (*p* = 0.76). The confusion matrix (Figure 10e) demonstrates a slight decrease in overall precision as the lack of personal metadata slightly reduces the model’s ability to contextualize physiological baselines. The corresponding feature importance (Figure 11e) reflects a shifting dominance back toward environmental T (0.21) when personal data is absent. Physiological parameters remain highly relevant, with Ts at 0.19 and Tf at 0.16, alongside CO_2_ (0.17), heart rate (0.15), and RH (0.13).

**Dataset 5: Reduced Sensor Monitoring Solution with Environmental, Physiological and Personal Parameters**

With Dataset 5, the feasibility of a low-cost, reduced sensor monitoring solution was tested utilizing a reduced set of parameters (T, heart rate) alongside personal data. The XGB model demonstrated highly balanced performance despite the reduced sensor count, achieving a mean *Accuracy* of 0.68 ± 0.2 (95% CI [0.6; 0.7]), an *F1-Score* of 0.68, a *Recall* of 0.68, and a *Precision* of 0.68. The strong predictive fit is clearly visible in the regression analysis (Figure 9c), which displays tight clustering. Feature importance (Figure 11c) highlights how personal characteristics step in to provide critical context. While T remains the dominant predictor (0.67), heart rate (0.14) and personal metrics like BMI (0.13) and age (0.07) provide substantial secondary explanatory power, whereas gender showed zero importance (0.00).

**Dataset 6: Bare-Minimum Configuration with Environmental and Physiological Parameters**

To test the absolute limits of this approach, personal data were subsequently removed in Dataset 6, which employed only T and heart rate. In this restricted setup, the XGB algorithm’s performance declined to a mean *Accuracy* of 0.60 ± 0.2 (95% CI [0.5; 0.6]), an *F1-Score* of 0.59, a *Recall* of 0.60, and a *Precision* of 0.59. Moving from this bare-minimum setup back to Dataset 5 highlights the value of personal parameters in low-sensor environments, resulting in a substantial +13.6% increase in accuracy. Crucially, a paired *t*-test confirms that this improvement is statistically significant (*p* = 0.01). The limitations inherent in this bare-minimum approach are evident in the linear regression (Figure 9f), which exhibits a noticeable deviation from the ideal fit characterized by a shallower slope. This is explicitly visible in the confusion matrix (Figure 10f): the model aggressively defaults to predicting ‘Comfort’, misclassifying extreme instances as neutral. The predictor importance analysis (Figure 11f) shows that T retains its status as the dominant factor with an importance score of 0.76, while heart rate provides the remaining explanatory power at 0.24.

## 4. Discussion

To address non-linearities in comfort perception, the study evaluated the performance of ML algorithms across varying data configurations using LOSO cross-validation, highlighting a complex trade-off between model complexity, input dimensions, and accuracy.

When contextualizing these findings within the broader landscape of PCMs, it is crucial to distinguish the proposed methodology from other individualized inference techniques. Prior studies have successfully utilized optimization methods, such as GA, to directly address individualized thermal-comfort modeling. These GA approaches have demonstrated exceptional performance, achieving 98% accuracy based on mean error evaluations and reducing Mean Square Error (MSE) to as low as 0.0005. These methods typically function by taking a baseline model (e.g., Fanger’s equations) and iteratively tuning unmeasurable coefficients like metabolic rate based on continuous feedback to learn a highly specific comfort profile tailored to a single occupant. While highly effective for individual-level inference, achieving near-perfect accuracies (e.g., 98%) frequently stems from optimizing a deterministic mathematical equation for a specific individual under controlled input assumptions. In contrast, the present multi-sensor ML approach represents a distinct methodological alternative. Nominally, the highest predictive accuracies achieved in this study, specifically the 73% realized by the SVM-LIN model (Dataset 2) and the 68% by the XGB model (Dataset 5), fall below the 98% benchmark of GA-optimized models. However, this variance highlights the transition from individual mathematical optimization to population-level generalization. Our framework does not reverse-engineer a unique theoretical equation for every new occupant; instead, it is entirely data-driven, bypassing theoretical equations to fuse static personal data (age, gender, BMI) directly with real-time, noisy physiological signals. This allows the model to naturally contextualize baseline physiological differences across a diverse group of users. As a result, it uses personal parameters to add valuable information to unseen occupants, offering a highly scalable, sensor-based alternative to strict occupant-specific optimization.

A notable finding of this study, reinforced by statistical testing, is the varied impact of personal data depending on the underlying sensor complexity. When comparing datasets with and without personal variables, the models exhibited distinct behaviors. In data-rich environments, whether relying on comprehensive environmental metrics (Dataset 2 vs. Dataset 1) or full physiological arrays (Dataset 4 vs. Dataset 3), the addition of personal data did not yield statistically significant changes in performance (*p* = 0.27 and *p* = 0.76, respectively). This suggests that complex sensor networks already capture the immediate manifestations of individual differences, rendering static personal data statistically redundant and occasionally introducing non-significant noise. However, as sensor complexity is intentionally minimized to simulate scalable, low-cost applications, personal data proves to be valuable information. Most strikingly, in the reduced sensor setup, adding personal data resulted in a highly significant +13.6% increase in accuracy (Dataset 6 vs. Dataset 5, *p* = 0.01), allowing the XGB model to increase predictive accuracy. When comparing the confusion matrices (Figure 10), without personal data in restricted setups, the models lack the demographic context required to interpret physiological thresholds, heavily under-predicting the extremes. Reintroducing personal data (Dataset 5) corrects this imbalance, restoring the model’s ability to accurately identify non-neutral thermal sensations. This proves that personal parameters add valuable information, especially when sensor networks must be minimized for real-world applications.

Furthermore, a critical insight from this modeling effort is the dynamic shift in feature importance, which varies significantly based on the available input variables (Figure 11). In the absence of physiological data, environmental factors, specifically T, completely dominated the model’s decision-making, showing overwhelming importance scores of 0.96 in Dataset 2 and 0.93 in Dataset 1. However, when physiological sensors were introduced in Datasets 3 and 4, the predictive influence exhibited a highly balanced distribution. The models prioritized the body’s physiological signals, with variables like Ts (up to 0.19) and Tf (up to 0.16) gaining significant weight alongside ambient conditions. Notably, in the reduced configurations, heart rate contributed significantly to the explanatory power, confirming that even a single physiological indicator provides essential information when comprehensive environmental data is scarce. The shift in heart rate importance between Dataset 5 (0.14) and Dataset 6 (0.24) does not undermine the value of personal data; rather, it reflects how the XGBoost algorithm dynamically reallocates weights. Because permutation importance is mathematically normalized to sum to 1, introducing static personal variables, such as BMI (0.13) and age (0.07), in Dataset 5 distributes the relative importance across a wider array of features. In Dataset 6, which represents a “bare minimum” application, heart rate acts as the only available physiological data, explaining its surge to a primary role. Crucially, the addition of personal data does not redundantly absorb physiological information. Instead, it provides distinct predictive value because personal variables establish a static thermoregulatory baseline (e.g., baseline metabolism and passive insulation), giving the algorithm the necessary context to accurately interpret the dynamic fluctuations captured by heart rate. Indeed, incorporating these personal parameters into the non-linear XGBoost model yielded an increase in accuracy. This definitively demonstrates that personal data provides independent and complementary context that dynamic heart rate alone cannot fully capture.

## 5. Conclusions

The measurement of IEQ is essential for occupant well-being, cognitive performance, and building energy efficiency. However, the state of the art in thermal comfort prediction has historically been limited by its reliance on Fanger’s model. Because it uses a one-size-fits-all methodology, the PMV model fails to account for human diversity and completely ignores critical personal data (gender, age, and BMI). By failing to account for these distinct physiological realities, such as the significantly different neutral temperatures between genders or the thermal insulation effects of varying body compositions, traditional models suffer from measurement errors. Consequently, when predicting the actual TSV of occupants in real-world scenarios, the PMV approach demonstrates an accuracy of less than 34%, highlighting a critical need to evolve from generic environmental conditioning to personalized measurement.

To address this profound limitation, this research demonstrates that leveraging ML offers a robust solution for thermal comfort prediction by systematically integrating personal, environmental, and physiological data evaluated through a LOSO cross-validation strategy. By evaluating various algorithms across distinct dataset configurations, this study highlights the critical value of personal data in thermal modeling.

Most notably, the results establish that personal data act as important contextual input when the sensor architecture is restricted. While its integration introduced non-significant noise in data-rich environmental models (a −4.7% variation in accuracy, *p* = 0.27) and yielded non-significant changes in comprehensive physiological setups (*p* = 0.76), it became an indispensable asset in bare-minimum sensor networks. In these reduced configurations, reintroducing personal data recovered critical predictive capabilities, boosting overall accuracy by a statistically significant +13.6% (*p* = 0.01).

When contextualizing these predictive performances against the broader state-of-the-art, a nuanced picture of thermal comfort prediction emerges. The recent literature highlights tree-based ensembles and advanced feature selection as the benchmark, with RF models utilizing SHAP and XGBoost models integrating metabolic and clothing data achieving peak predictive accuracies between 90% and 96.7%. Similarly, deep learning approaches, such as LSTM networks, have reached approximately 90% accuracy when modeling temporal comfort dynamics. Nominally, the highest predictive accuracies achieved in this study, specifically the 73% realized by the SVM-LIN model (Dataset 2) and the 68% by the XGB model (Dataset 5), fall below these upper-tier benchmarks. However, this variance does not indicate a methodological shortcoming, but rather highlights the fundamental transition from controlled environments to real-world deployment. The near-perfect accuracies reported in the state-of-the-art studies frequently rely on heavily engineered static datasets, explicit manual inputs (e.g., precise clothing insulation values), or data collected in highly controlled climatic chambers without strict participant-independent validation protocols. In contrast, this study leverages raw, continuous data streamed from wearable sensors and indoor environmental sensors in an active, dynamic office environment, validated strictly on unseen participants. This approach inherently introduces natural noise, physiological artifacts from daily occupant movement, and non-linear complexity. Therefore, our results represent a highly realistic performance threshold for continuous, low-intervention monitoring in actual operational buildings. Crucially, even amidst this real-world noise, the integration of personal data provided a decisive performance baseline boost in low-sensor configurations, definitively validating the core premise of PCMs.

Furthermore, the univariate data clearly reflected the physiological disparities noted in the literature, confirming that females generally experience colder sensations in identical environments compared to males. Feature importance analysis revealed a dynamic shift in predictive drivers based on input dimensionality: while T overwhelmingly dictates model behavior in standard environmental datasets (with importance scores up to 0.96), direct physiological measurements (e.g., Ts up to 0.19 and Tf up to 0.16) create a highly balanced predictive distribution when introduced. The innovative part of this study lies in its deployment of a dual-sensor approach, monitoring both physiological signals and environmental conditions, while actively integrating subjective feedback and personal data. In practical real-world cases, this methodology increases the prediction accuracy of TSV. Notably, while the bare-minimum sensor configuration (using only ambient T and heart rate) achieved an accuracy of 60%, reintroducing static personal parameters (such as BMI and age) provided the algorithm with the thermoregulatory context needed to interpret dynamic heart rate fluctuations, remarkably boosting the accuracy to 68%. This still significantly outperforms the traditional PMV model (34% accuracy), validating the feasibility of low-cost, real-time thermal monitoring systems, provided they respect and integrate individual physiological differences.

Finally, the validation of the GSV clearly demonstrates that when subjects achieve optimal thermal comfort (TSV = 0), they experience significantly higher overall global comfort (a GSV approaching 10). This proves that maximizing an occupant’s thermal comfort is a direct pathway to maximizing their overall global comfort, reinforcing the practical value of personalized, real-time thermal management.

Future work will address key challenges that currently hinder widespread real-world deployment. First, while this study relied on a relatively small sample limited to young adults, expanding the participant pool is essential. A larger dataset is not only necessary to include older age groups and vulnerable populations, but it is also critical from a Machine Learning modeling perspective. Currently, the restricted sample size necessitates the use of robust linear baseline models (such as SVM-LIN) to prevent severe overfitting. However, linear algorithms evaluate independent, additive contributions, limiting their ability to map complex interaction effects, such as gender-specific thermal responses that only manifest at specific temperature extremes. By expanding dataset size and diversity, future work will enable the reliable deployment of complex algorithms capable of isolating and predicting these nuanced, demographic-specific thermal responses. Pending future validation across broader demographics, this methodology suggests a promising prospective application for vulnerable categories, demonstrating how intelligent building systems might eventually adapt to actual, individual human needs rather than estimated statistical averages. Furthermore, exploring this approach for older demographics could offer a non-invasive solution for individuals who may have less familiarity with technology by reducing or entirely avoiding the need for continuous, active user feedback. Second, because continuous wearable sensors inevitably introduce noise and artifacts due to daily occupant movements, future research must prioritize advanced data filtering and robust feature engineering to improve model reliability in uncontrolled environments. Third, while all data in this study was handled with strict anonymity, the future commercial scaling of PCMs must account for user privacy regarding the continuous transmission of physiological data to cloud servers. Therefore, near-term efforts will focus on developing lightweight models for real-time edge computing, enabling local data processing directly at the sensor level to intrinsically preserve user privacy and trust. Finally, to advance the primary objective of this paper, developing adaptive data-driven models for personalized comfort, future research will consolidate and expand this multi-sensor approach through specific measurement campaigns designed to capture holistic indoor environmental quality. While the current study utilized the GSV exclusively for descriptive statistical validation, these subsequent campaigns will aim to train advanced ML architectures to directly predict the GSV, bridging the gap between thermal neutrality and overall occupant well-being.

## Figures and Tables

**Figure 1:**
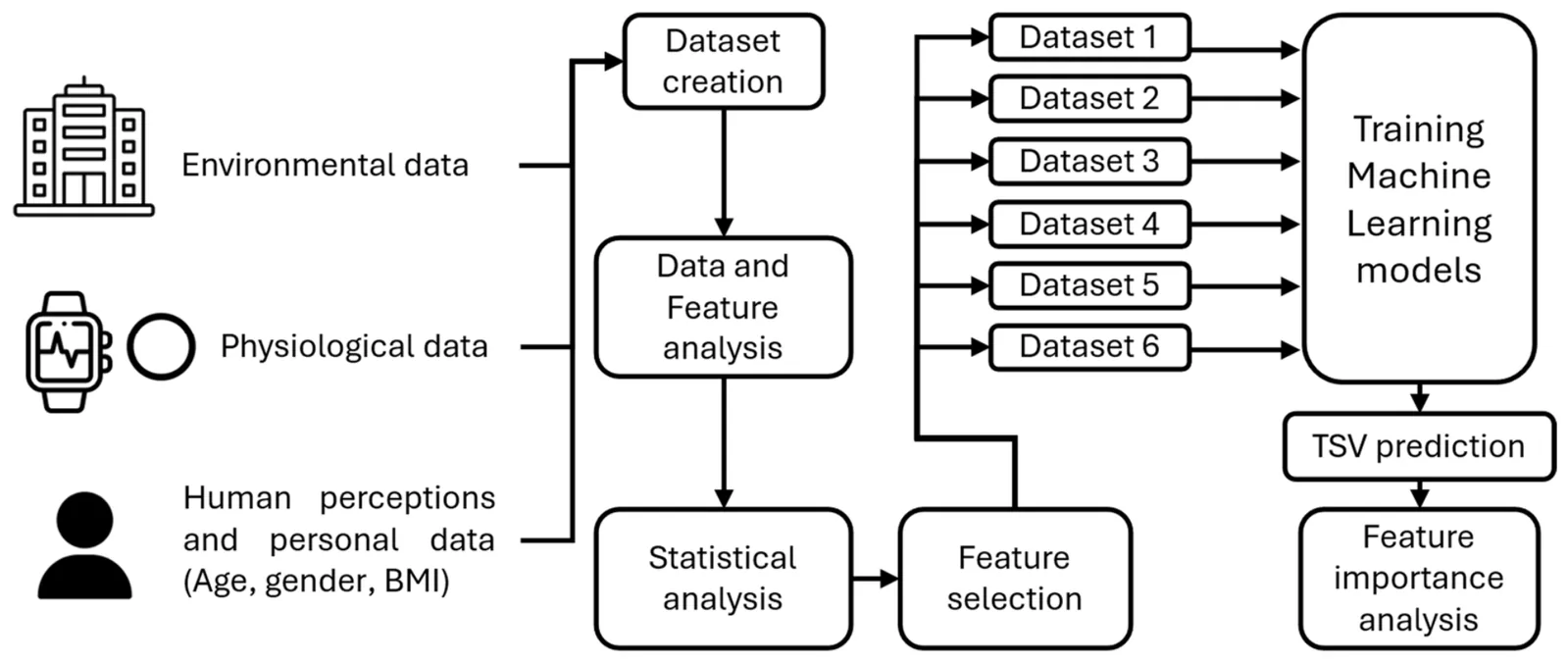
Adopted process for the TSV prediction.

**Figure 2:**
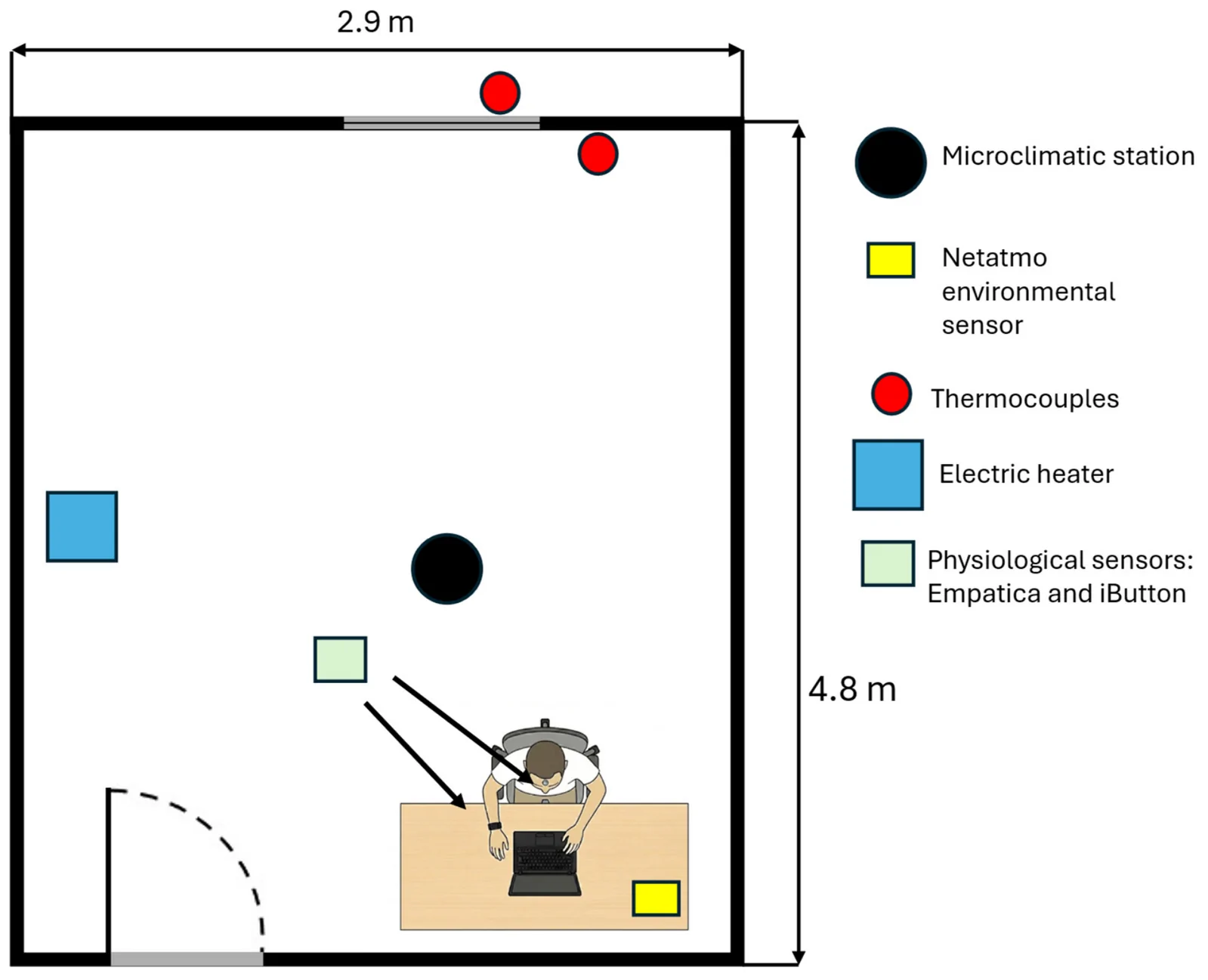
Experimental room layout and instrumentation.

**Figure 3:**
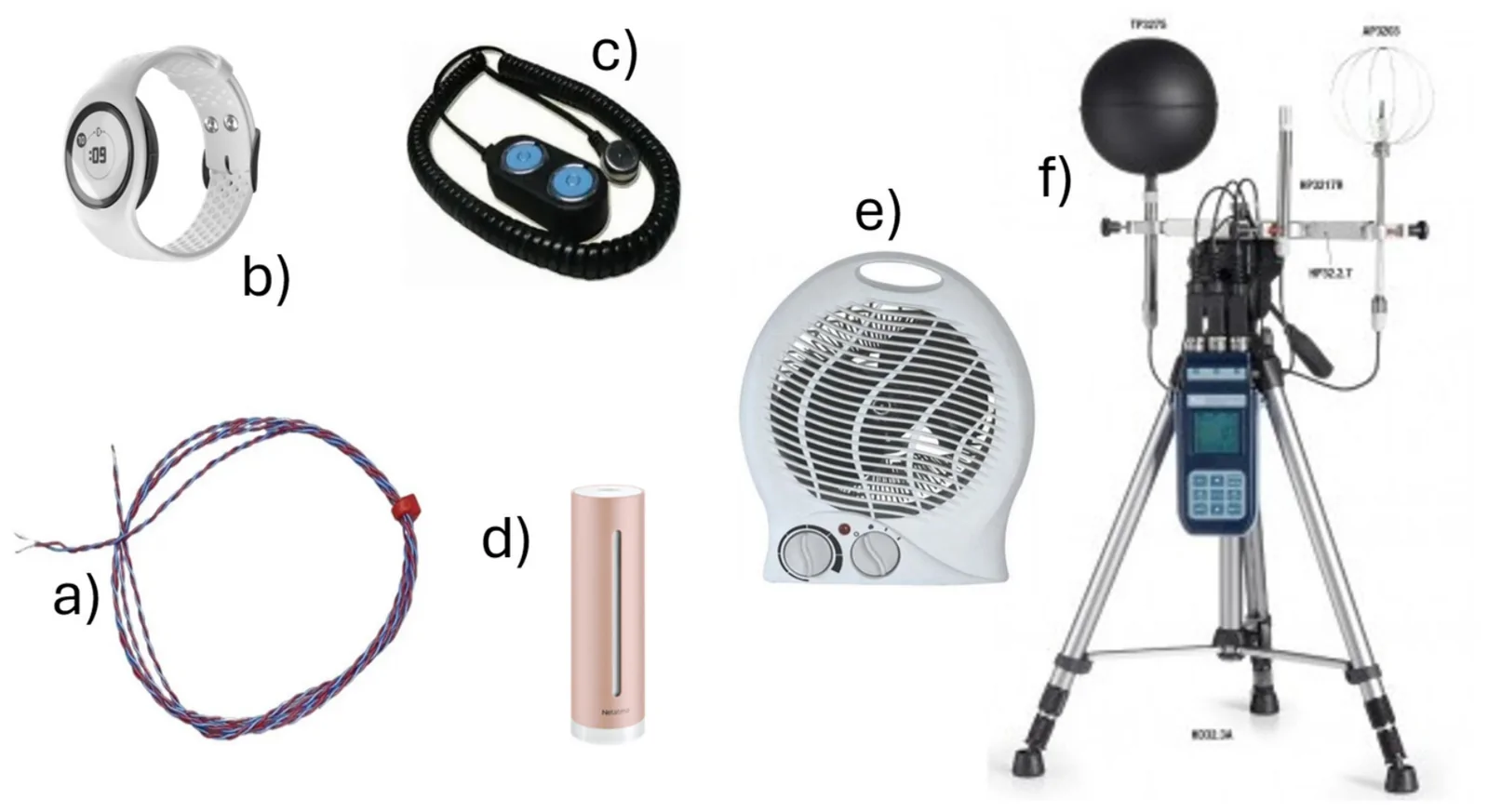
Instrumentation adopted during the measurement campaign: (**a**) Thermocouples, (**b**,**c**) physiological sensors (iButton and Empatica), (**d**) Netatmo monitor, (**e**) electric fan, and (**f**) microclimatic station.

**Figure 4:**
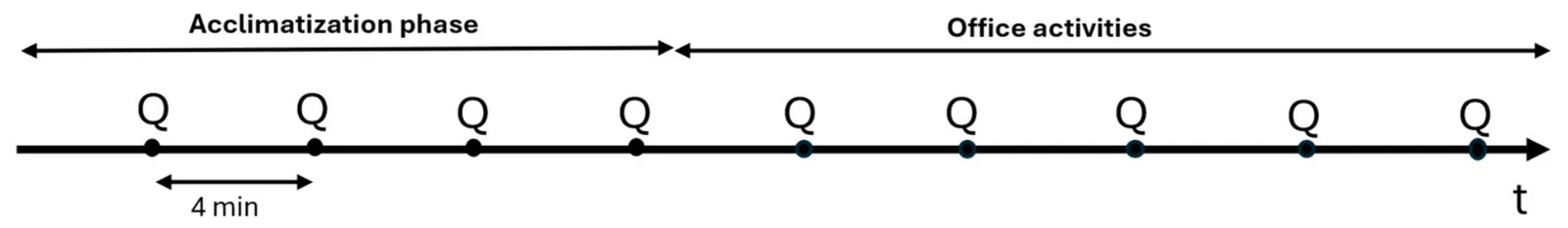
Schematic timeline of the carried-out test. Q stands for the provided questionnaire.

**Figure 5:**
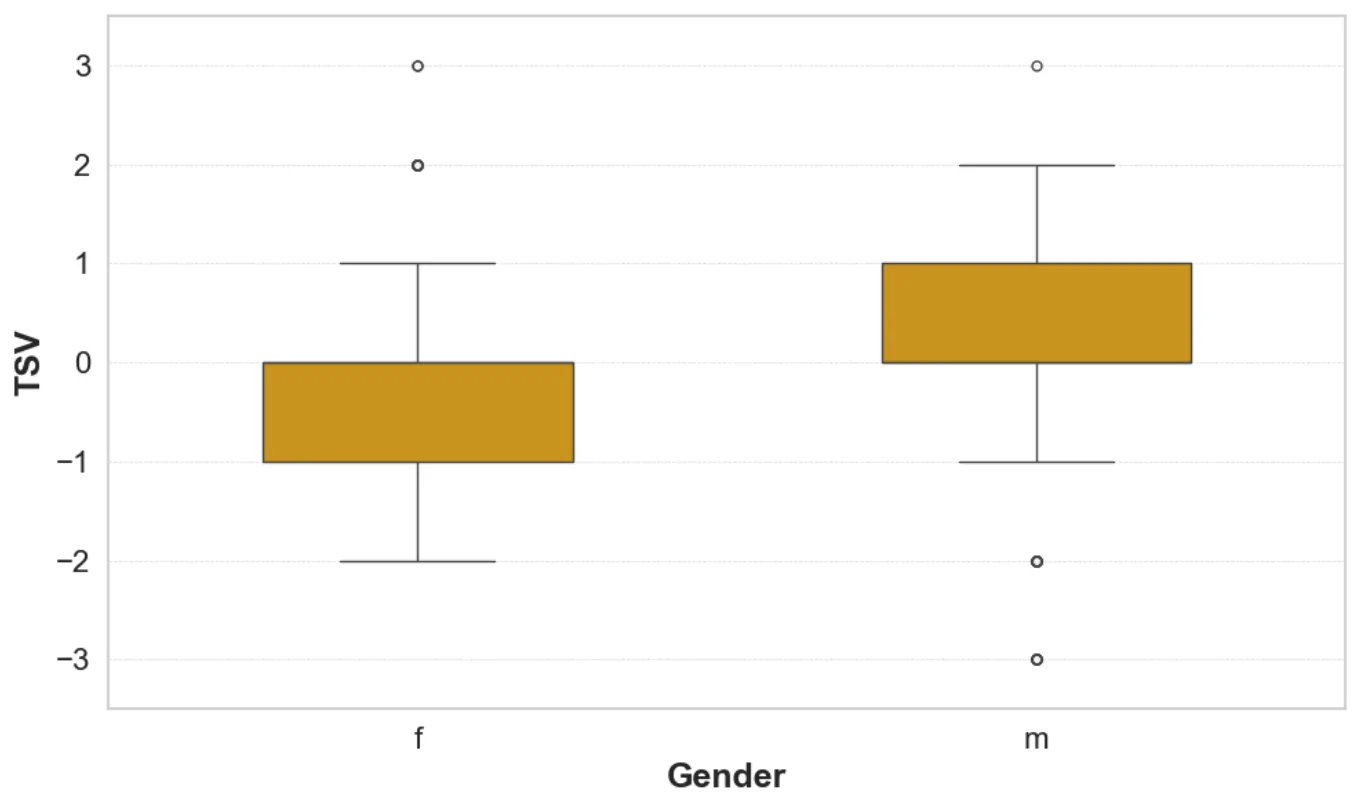
Comparison of TSV with gender.

**Figure 6:**
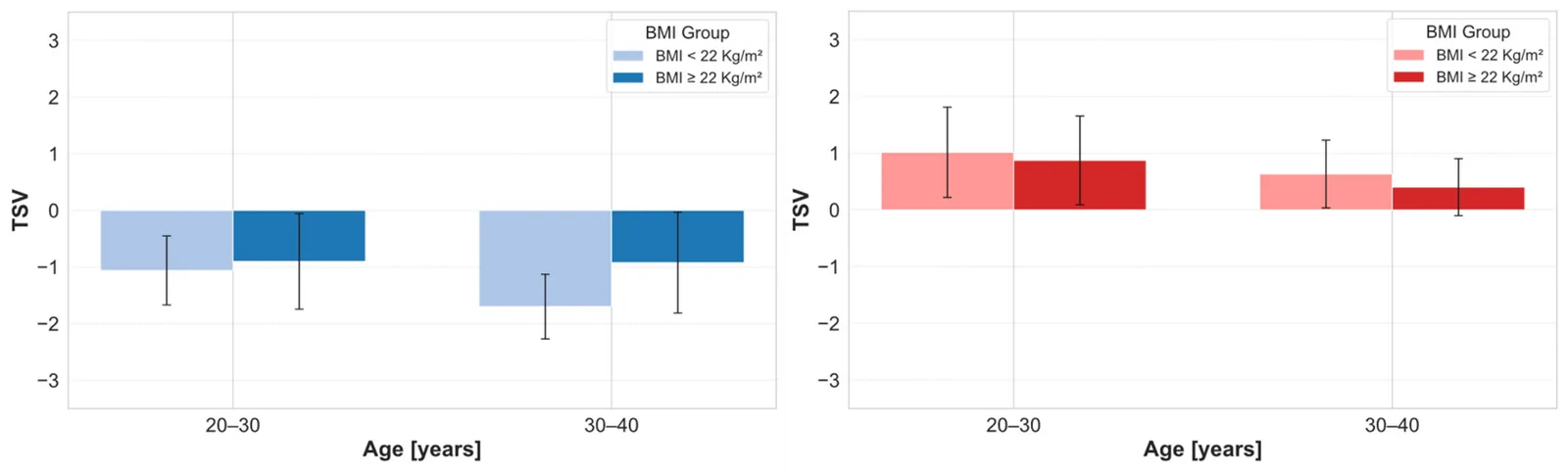
On the (**left**), TSV compared with age and BMI for the cold trial. On the (**right**), TSV compared with age and BMI for the hot trial.

**Figure 7:**
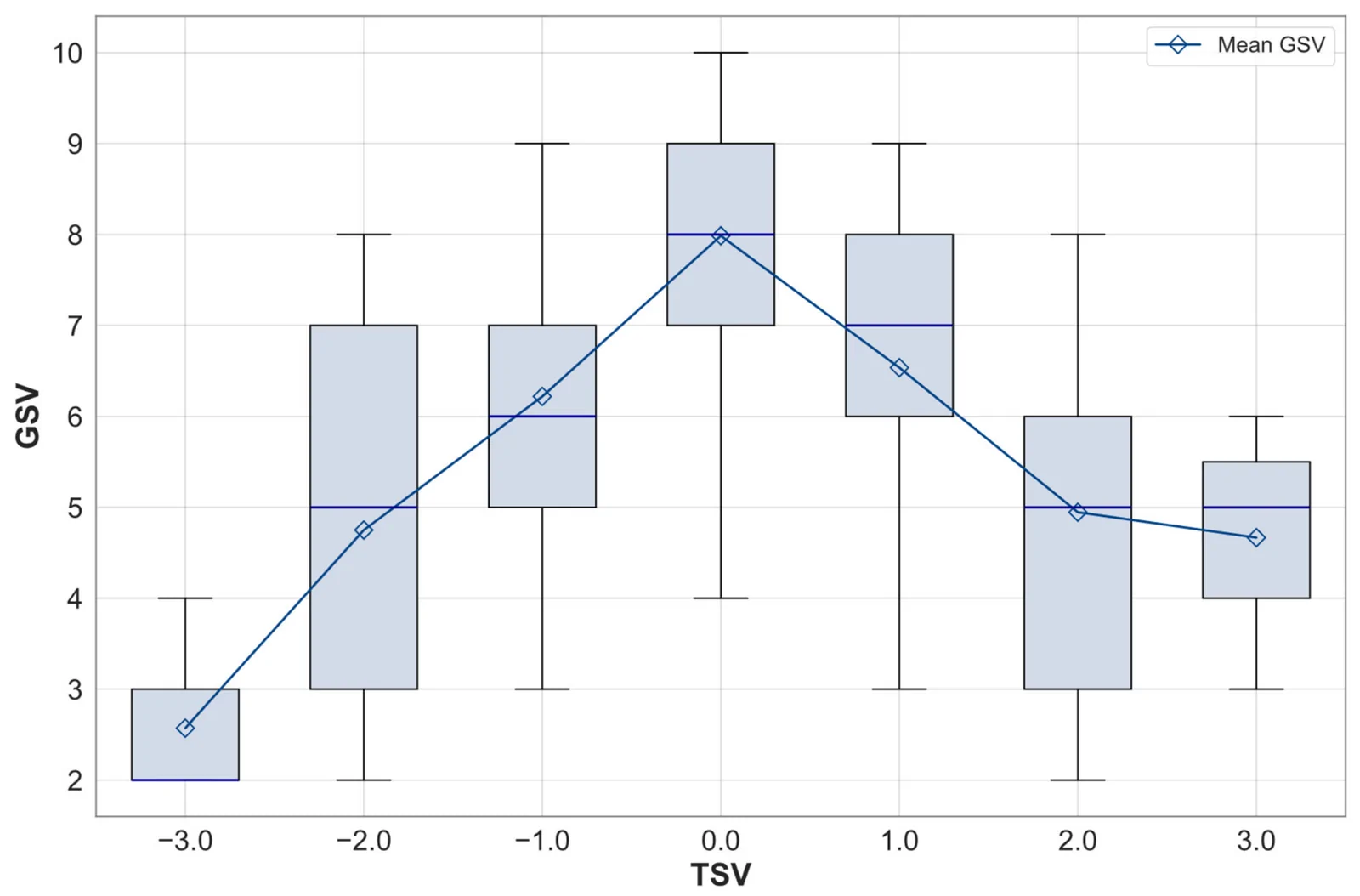
Statistical analysis of the relationship between TSV and GSV. The box-plots illustrate the variability of GSV data across discrete TSV intervals. The hollow diamonds connected by the solid line represent the mean GSV value, highlighting the central trend relative to the perceived thermal sensation vote.

**Figure 8:**
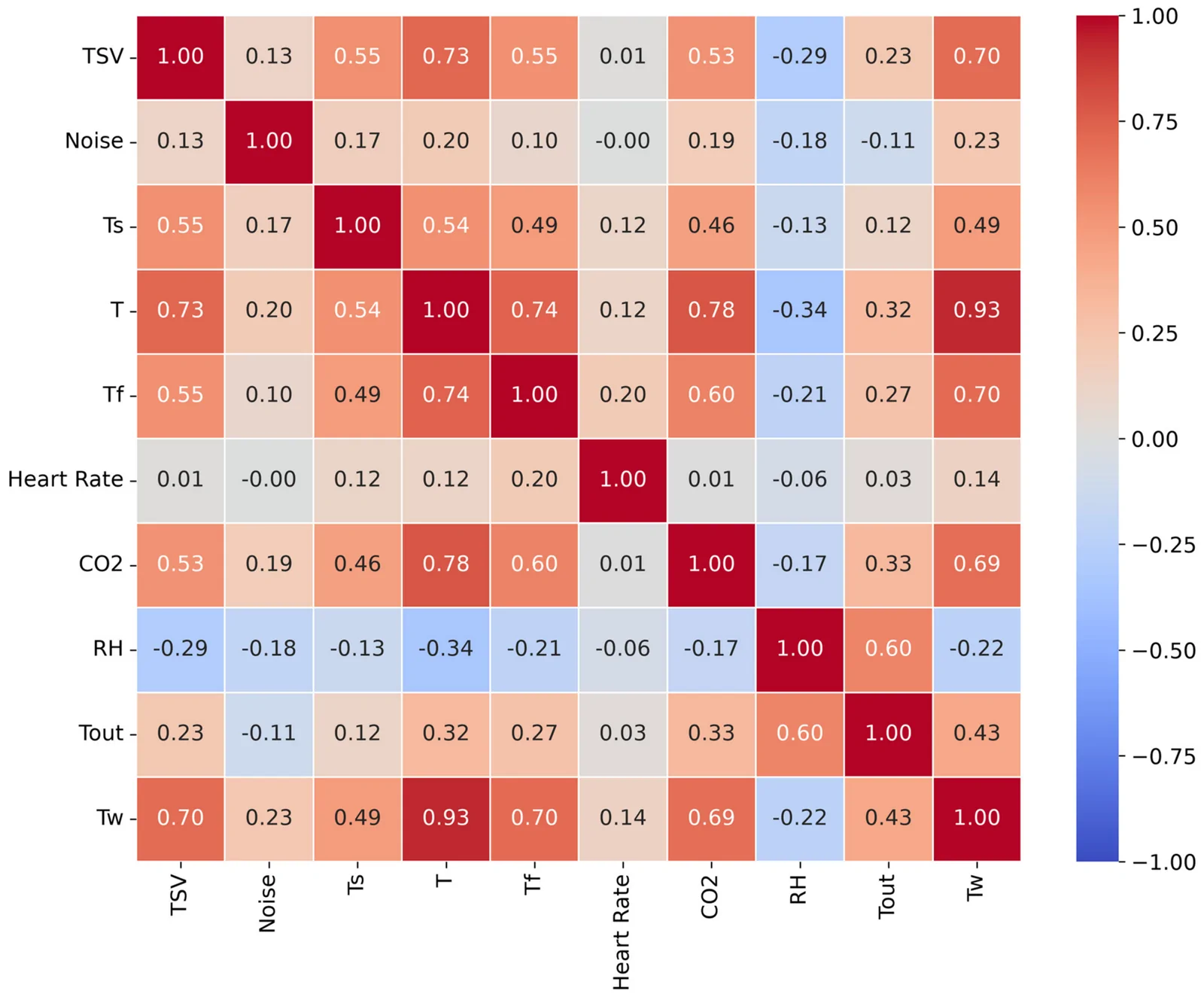
Pearson’s correlation matrix.

**Figure 9:**
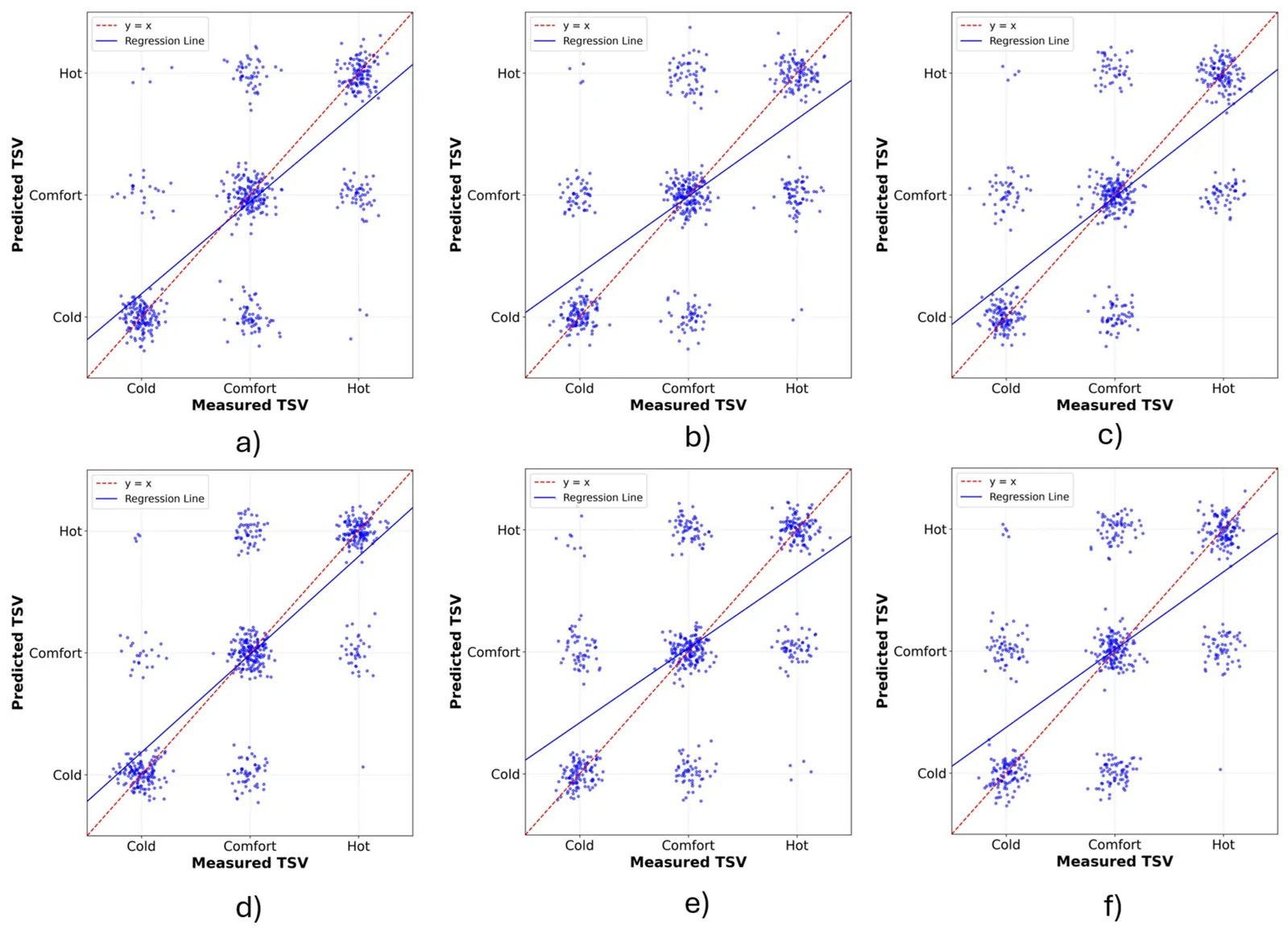
Comparison between measured TSV and predicted TSV: Linear regression analysis for the top-performing algorithms across the evaluated data configurations: (**a**) SVM-LIN algorithm (Dataset 1), (**b**) KNN algorithm (Dataset 3), (**c**) XGB algorithm (Dataset 5), (**d**) SVM-LIN algorithm (Dataset 2), (**e**) KNN algorithm (Dataset 4), and (**f**) XGB algorithm (Dataset 6).

**Figure 10:**
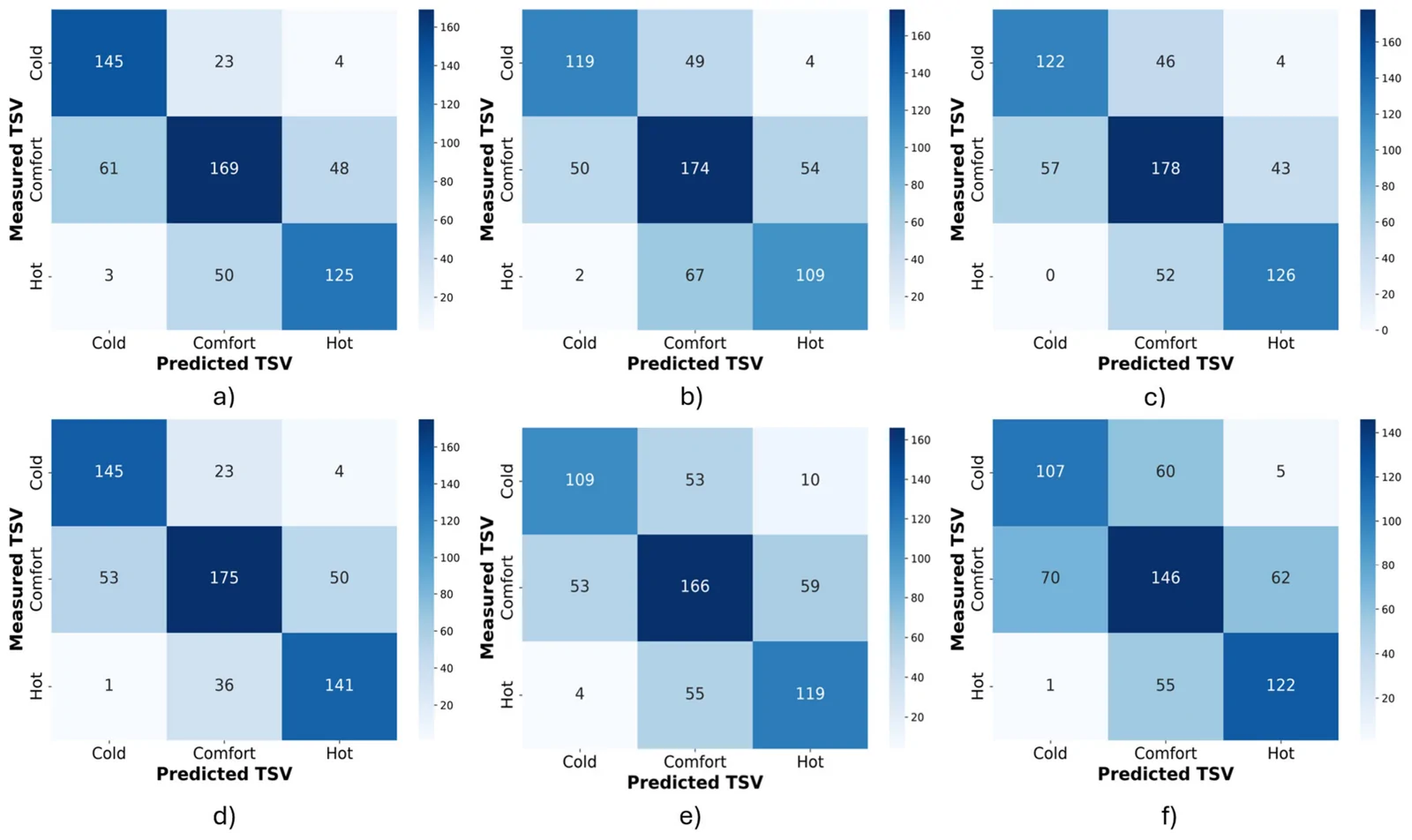
Comparison of confusion matrices across the six evaluated data configurations: (**a**) Dataset 1, (**b**) Dataset 3, (**c**) Dataset 5, (**d**) Dataset 2, (**e**) Dataset 4, and (**f**) Dataset 6, for the top-performing ML models.

**Figure 11:**
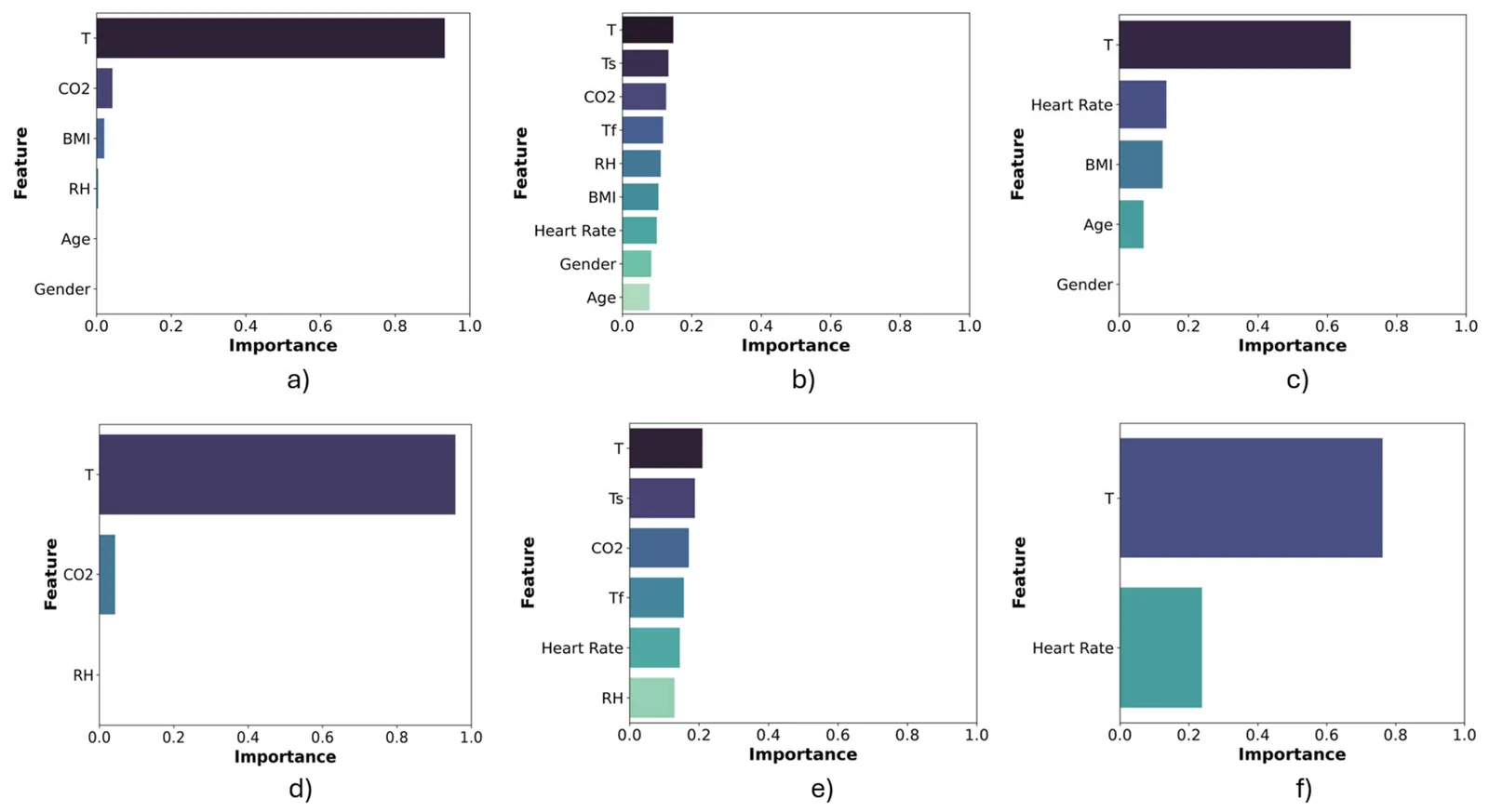
Comparison of predictor importance across the six evaluated data configurations: (**a**) Dataset 1, (**b**) Dataset 3, (**c**) Dataset 5, (**d**) Dataset 2, (**e**) Dataset 4, and (**f**) Dataset 6.

**Table 1:** Sensor datasheets.

Sensors	Measured Parameter	Measurement Range	Resolution	Accuracy
Netatmo Smart Indoor Air Quality Monitor	T	0 ÷ 50 °C	0.1 °C	±0.3 °C
	RH	0 ÷ 100%	1%	±3% RH
	Noise	35 ÷ 120 dB	0.1 dB	±3 dB
	CO_2_ concentration	0 ÷ 5000 ppm	1 ppm	±50 ppm
Empatica Embrace Plus	Pulse Rate	30 ÷ 240 bpm	1 bpm	±3 bpm
	HRV	0 ÷ 300 ms	0.01 ms	98% of positive agreement
	Ts	0 ÷ 50 °C	0.1 °C	±0.1 °C
iButton	Tf	−40 ÷ 85 °C	0.5 °C	±1 °C
Termocouples	Tw, Tout	−200 ÷ 350 °C	0.1 °C	±0.5 °C
Delta OHM HD32.1 Microclimatic Station	T	−20 ÷ 80 °C	0.1 °C	±0.2 °C
	RH	0 ÷ 100%	0.1% RH	±2% RH

**Table 2:** List of the collected parameters.

Environmental Parameters	Personal Parameters	Physiological Parameters
CO2, RH, T, Noise, T_out,_ T_w_	Age, gender, weight, height.	Tf, Ts, Heart Rate

**Table 3:** Hyperparameters adopted per ML model.

ML Model	Hyperparameters
Gradient Boosting Classifier (GBC)	*n_estimator* = 100 *learing_rate* = 0.1 *random_state* = 42
Random Forest Classifier (RF)	*n_estimator* = 100 *max_depht* = 10 *random_state* = 42 *n_jobs* = −1
Multiple-Layer Perceptron Classifier (NN)	*hidden_layer_sizes* = (32, 16) *max_iter* = 500 *alpha* = 0.001 *random_state* = 42
Support Vector Machine (SVM-RBF)	*kernel* = rbf *C* = 1.0 *gamma* = ‘scale’ *random_state* = 42)
Support Vector Machine (SVM-LIN)	*kernel* = linear *C* = 1.0 *random_state* = 42
Naive Bayes (NB)	Default parameters
Logistic Regression (LR)	*C* = 1.0, *random_state* = 42 *max_iter* = 1000 *n_jobs* = −1
XGBoost (XGB)	*n_estimators* = 100 *max_depth* = 6 *eval_metric* = mlogloss
LightGBM (LGBM)	*n_estimators* = 100 *num_leaves* = 31

**Table 4:** Dataset configuration with a focus on input parameters.

	Input Data		Target Value
**Dataset 1** Environmental, Personal data	**Environmental parameters**	CO_2_, RH, T	**TSV**
	**Personal Parameters**	Age, Gender, Weight, Height	
**Dataset 2** Environmental	**Environmental parameters**	CO_2_, RH, T	**TSV**
**Dataset 3** Environmental, Personal, Physiological data	**Environmental parameters**	CO_2_, RH, T	**TSV**
	**Personal Parameters**	Age, Gender, Weight, Height	
	**Physiological parameters**	Tf, Ts, Heart Rate	
**Dataset 4** Environmental, Physiological data	**Environmental parameters**	CO_2_, RH, T	**TSV**
	**Physiological parameters**	Tf, Ts, Heart Rate	
**Dataset 5** Reduced environmental, reduced physiological data, personal data	**Environmental parameters**	T	**TSV**
	**Personal Parameters**	Age, Gender, Weight, Height	
	**Physiological parameters**	Heart Rate	
**Dataset 6** Reduced environmental and physiological data	**Environmental parameters**	T	**TSV**
	**Physiological parameters**	Heart Rate	

**Table 5:** ML results for best-performing models.

	Best Model					
Input Dataset	*Accuracy* (Mean ± SD)	*F1-Score*	*Recall*	*Precision*	95% CI	*p*-Value
	**SVM-LIN**					
**Dataset 1**	0.69 ± 0.2	0.69	0.70	0.70	[0.6; 0.7]	0.27
	**SVM-LIN**					
**Dataset 2**	0.73 ± 0.2	0.73	0.73	0.73	[0.6; 0.8]	/
	**KNN**					
**Dataset 3**	0.64 ± 0.2	0.64	0.64	0.64	[0.5; 0.7]	0.76
	**KNN**					
**Dataset 4**	0.63 ± 0.2	0.63	0.63	0.63	[0.5; 0.7]	/
	**XGB**					
**Dataset 5**	0.68 ± 0.2	0.68	0.68	0.68	[0.6; 0.7]	0.01
	**XGB**					
**Dataset 6**	0.6 ± 0.2	0.59	0.60	0.59	[0.5; 0.6]	/

## Data Availability

The data presented in this study are available on request from the corresponding author.
